# Molecular Mechanism and Targeted Therapy Options of Triple-Negative (ER, PgR, HER-2/neu) Breast Cancer: Review

**DOI:** 10.4021/wjon681e

**Published:** 2013-07-15

**Authors:** Mahesh Kandula, Kalyan Kumar Ch, Ammi Raju YS

**Affiliations:** a Krisani Biosciences Private Limited, Alexandria, Genome valley, Hyderabad, India

**Keywords:** Trriple negative breast cancer, EGFR, Metastasis, BRCA1, Inhibitors, PARP

## Abstract

Tripple negative breast cancer (TNBC) accounts for approximately 15% of breast cancers. It is defined by the absence of estrogen receptor (ER), progesterone receptor (PR), and HER-2 Over expression. Expression of ER, PR and HER-2 plays an important role in therapeutic assessment of patients with breast cancer. TNBC is not one disease, but a family of diseases, some of which are highly aggressive with limited treatment options. Triple-negative breast cancers Patients are not benefiting from currently available receptor-targeted systemic therapy. At present, there is no single agent that targets triple-negative breast cancer. However, researchers are presently investigating large number of potential therapies that may eventually improve outcomes in these patients. In this review article, we discussed about tripple negative breast cancer, also the role of BRCA gene mutations and targeted therapeutic options available to triple negative breast cancer patients.

## Introduction

Triple-negative breast cancers are characterized by the absence of estrogen receptor, progesterone receptor and human epidermal growth factor receptor [1-3]. An estimation of 1 million cases of breast cancer is diagnosed annually worldwide. Of these, approximately 170,000 are of the triple-negative (ER-/PR-/HER2-) phenotype [4]. TNBC accounts for approximately 15% of breast cancers [5]. Triple-negative disease is diagnosed more frequently in premenopausal patients (< 50 years), are more prevalent in African-American women. In random cohort studies of 148 Nigerian patients, 66.9% were premenopausal women with a mean age of 43.8 years when diagnosed with triple negative tumors [6]. Triple negative breast cancers are more aggressive than other sub types of breast cancer with larger tumor size, higher grade and showing lymph node involvement which has a distant metastasis-free survival rate of 71% for a period of 5 years [7]. TNBC is an important area of research for both researchers and clinicians alike because TNBC is a poor prognostic factor for disease-free and overall survival, no effective specific targeted therapy is readily available for TNBC.

## TNBC Biology

Multiple studies of gene expression profiling have advanced the understanding of the molecular diagnosis of breast cancer. Perou et al were the first to describe the various molecular subtypes or molecular profiles of breast cancers [8]. More recently, gene expression analysis using DNA microarray technology has identified additional breast tumor subtypes. They are Luminal A, luminal B, normal breast- like, Her2 over expression and Basal-like, each subtype with different prognosis. Basal-like tumors originate in the outer cells that line the mammary ducts. Basal-like breast cancers with a triple negative phenotype are termed as triple negative breast cancer characterized by the increased expression of high molecular weight basal markers like CK 5/6, CK 17, EGFR, CK 14 [9]. Their incidence has been estimated to be between 13% and 25%. Triple-negative breast cancer with the expression of basal markers (basal like) shows an aggressive nature of the disease when compared with the Triple-negative breast cancer without the expression of these basal markers [10]. Women with early stage Triple-negative breast cancers are associated with poor Nottingham prognostic index, develop recurrence, metastasis and poor survival. While the TNBC phenotype is defined by immunohistochemistry, no established diagnostic criteria have been identified for basal-like breast cancer on a morphological basis. From a pathologist’s point of view, triple-negative tumors and basal-like tumors are predominantly of high histologic grade and poorly differentiated when examined morphologically [11]. It is important to realize that TNBC and basal-like breast cancer are not all of the high histological grade.

## TNBC Metastasis and Recurrence

Metastasis, the major cause of mortality in patients with breast cancer, is caused by tumor cells that escape from the primary tumor into the bloodstream and travel through the circulation to distant sites where they develop into secondary tumors [12]. Triple-negative breast cancer patients have a high risk of recurrence and death when compared with other types of breast cancer patients. A previous study has reported that there is a high risk of lung and brain metastasis due to the first site of recurrence in Triple-negative breast cancer patients [13]. Patients with metastasis Triple negative breast cancer have a risk (6-46%) of central nervous system metastasis [14, 15], the risk of CM was especially observed in young patients with node positive disease [15]. Triple-negative breast cancer patients with Central nervous system replase had a lower survival of 2.9 months of the first site and 5.8 months at the latter site [16]. Early stage Triple-negative breast cancer patients with RD (residual disease) treated with Neoadjuvant chemotherapy have worse survival rates, however patients with higher PCR rates (pathological complete remission) after Neoadjuvant chemotherapy have a better survival rate but with poor prognosis compared to that of other types of breast cancer. Triple-negative breast cancer patients with no pathological complete remission rates are at a higher risk of developing recurrences [17]. The majority of the triple-negative breast cancer patients had a high expression of EGFR, VEGF and Ki 67 had a poor prognosis and shorter survival. Triple-negative breast cancers with lower expression of Androgen receptor, P53 and E-cadherin were observed with a higher histological grade and it leads to recurrence and metastasis [18-20].

## Relation Between TNBC and BRCA1

Hereditary breast cancers account for only 5-10% of all breast cancer cases. The functions of BRCA1 are the repair of double stranded DNA breaks by the potentially error-free mechanism of homologous recombination. Lack of BRCA1 could result in DNA repair by more error-prone mechanisms such as nonhomologous end-joining and single-strand annealing, resulting in genomic instability and therefore cancer predisposition [21] However, individuals carrying mutations in the BRCA gene have 40-80% chance of developing breast cancer. Thus, identification of BRCA mutations has been used as one of the strongest breast cancer predictors. Mutations in the BRCA1 and BRCA2 genes occur with different frequencies in individuals of different ethnicities living in different geographic regions in the world. Interestingly, DNA microarray and immunohistochemical analyses revealed that 80-90% of breast cancers in women with germ-line mutations in BRCA1 are triple-negative [22]. Mutations in the BRCA1 gene have been demonstrated to lead to error-prone DNA repair, resulting in genomic instability and thus predisposition to carcinogenesis. Several in vitro studies have indicated that breast tumor cells with BRCA1 mutations are extremely sensitive to drugs that induce cross links (mitomycin-C and platinum) and single- and double-strand breaks (etoposide and bleomycin) in DNA. Studies have shown that breast cancers in women with germ-line BRCA1 mutations are more likely to be triple-negative and high-grade. The majority of tumors arising in BRCA1 germ-line mutation carriers, in particular those diagnosed before 50 years of age. Most BRCA1-associated tumors are triple-negative, and the patients in which they arise have a poor outcome. In some triple negative tumors of high histologic grade, brca1 protein levels have been shown to be significantly lower, suggesting that the brca1 pathway may be dysfunctional in these tumor cells. Other mechanisms resulting in downregulation of BRCA1/2, including epigenetic alterations and overexpression of BRCA1 inhibitors are also associated with TNBC

## Therapy Options for TNBC

Triple-negative breast cancer Patients do not benefit from hormonal or trastuzumab-based therapies because of the loss of target receptors such as ER, PR, and Her-2. Hence, surgery and chemotherapy, individually or in combination, appear to be the only available modalities. Chemotherapy improves the outcome to a greater extent when used in patients with triple-negative breast cancer than when used in patients with the much more common ER-positive subtype. Currently, there is no preferred standard form of chemotherapy for triple-negative breast cancer. Chemotherapy Drugs for example carboplatin, cisplatin, parp1 inhibitors, and docetaxel could be very useful in the administration of patients with advanced triple-negative cancers. The addition of docetaxel or paclitaxel to anthracycline-containing adjuvant regimens may be of greater benefit for the treatment of ER-negative and HER2-negative cancers than for the treatment of ER-positive, HER2-negative cancers, which are much more common [23]. To improve outcomes of TNBC, we must unravel its biological pathways and modes of progression and use that knowledge to develop novel targets and therapies.

### Drugs for PARP inhibitor

The most interesting clinical target in triple-negative breast cancer is the enzyme poly (adenosine diphosphate- Ribose) polymerase (PARP), which is involved in base-excision repair after DNA damage. Several PARP1 inhibitors are currently in clinical development and hold promise in TNBC. PARP inhibitors have recently shown very encouraging clinical activity in early trials of tumors arising in BRCA mutation carriers. One of these inhibitors, iniparib (BSI-201), which was recently used in a randomized phase 2 trial involving patients with triple-negative cancer. When this inhibitor was added to a chemotherapy combination of gemcitabine and carboplatin, there were significant improvements in the rate of tumor regression. A phase II study with a combination of Carboplatin and Gemcitabine in metastatic triple-negative patients determined the activity in response rates from 26% to 34%. This study was conducted to observe if Iniparib could effectively increase the antiproliferative and cytotoxic nature of the two chemotherapeutic drugs which gave a positive output [24-26]. The combination of Iniparib along with Gemcitabine and carboplatin demonstrated an increase in efficiency in terms of clinical benefit, overall survival, progression free survival, and the rate of complete or partial response in metastatic Triple-negative breast cancer patients [27]. Similarly, the use of an oral PARP inhibitor, olaparib, often after chemotherapy had failed, resulted in tumor regression in up to 41% of patients carrying BRCA mutations, most of whom had triple-negative breast cancer [28]. In both instances, these benefits were achieved with minimal toxicity. Previous observations provided strong circumstantial evidence that the brca1 and parp1 pathways could be dysfunctional in a significant subgroup of triple-negative and basal-like breast tumors and, therefore, that those pathways could be targeted for therapy 60, 63 tumors which lack BRCA1 are sensitive to the PARP inhibitors which result in synthetic lethality [26]

### Drugs for EGFR inhibitor

EGFR inhibition in breast cancer has been another interesting story. The EGFR signaling pathway is another area of investigation in TNBC, since EGFR expression may be more apparent in basal-cell type breast cancers. EGFR is expressed approximately in 60% of the triple negative breast cancer and is a marker of poor prognosis irrespective of the lymph node involvement and tumor size and may also contain mutations in the p53 gene [29]. Due to its prognostic and diagnostic role in basal like Triple negative breast cancer many drugs have been employed to inhibit its action [30]. Cetuximab a chimeric monoclonal antibody was employed to target EGFR and showed a lower response in an advanced stage Triple negative breast cancer. Inhibition of EGFR might be a useful therapeutic strategy. Several studies of cetuximab with or without carboplatin or irinotecan/carboplatin suggested potential improvements in partial response of metastatic TNBC [31].

### Drugs for mTOR inhibitors

The mTOR (mammalian target of rapamycin) is associated with cell cycle regulation and an effector of the common pathway of phosphatidylinositol 3-phosphate phosphatase and PTEN/AKT pathway. Breast cancers are associated with the impairment of this pathway. Loss of PTEN tumor suppressor gene is a common event in triple negative breast cancers which also leads to the activation of mTOR [32]. As a result many inhibitors are employed to inhibit mTOR activation. A phase II randomized study with a setting of the first line or second line treated 59 metastatic breast cancer patients was used to evaluate two everolimus (oral mTOR inhibitor) regimens, one with 10 mg/day and the second regimen with 70 mg/week. The response rate of 12% and 0% was observed in daily regimen vs weekly regimen respectively. A high risk of pneumonitis was seen in a daily regimen when compared with the weekly regimen (6%) were observed [33]. A phase II nonrandamized study with triple negative breast cancer patients is currently evaluating the temsirolimus, an intravenous mTOR inhibitor and a phase III randamized study with a neoadjuvant setting is currently evaluating everolimus in combination with antracyclines and taxanes [34].

### Drugs for Src tyrosine kinase inhibitors

High expression of src has been observed in breast cancer tissues compared to that of the normal breast samples indicating an activation of src kinase in breast cancer tissues. Activation of src is associated with the activation of EGFR pathway which is frequently expressed in triple negative breast cancers [35]. Due to the significant role of Src in growth, proliferation, invasion, angiogenesis and metastasis has rationalized the need for the development of src inhibitors in breast cancer. Inhibition of src may reduce recurrence and metastasis in the residual disease and also slow down the disease progression. As src is associated with osteoclast function it is an important target of patients with metastatic breast cancer. Impairment of src pathway in osteoclast results in the improper formation of ruffled membrane which is an important event in bone resorption [36]. Dasatinib is an oral tyrosine kinase inhibitor which acts on src and abl proteins. A phase II study with dasatinib in metastatic triple negative breast cancer patients showed a clinical benefit rate of 9%. However the results were not significant due to discontinuation of the therapy and low dose levels [37].

## Conclusion

Tripple negative breast cancer is harder to treat as the tumors don’t have the receptors that the common drugs such as oestrogen, progesterone or HER2 that are targeted by common treatments such as hormone therapy or Herceptin can target blocking the growth of the tumor. Breast tumor cells with BRCA1 mutation carriers may have particular sensitivity to platinum agents and relatively less sensitivity to taxanes. Therefore, the identification of novel molecular biomarkers to predict response to specific chemotherapy is required to further improve treatment strategies.
